# Electroacupuncture alleviates osteoarthritis and is associated with activation of the Nrf2/HO-1 pathway and reduced ferroptosis-related changes

**DOI:** 10.3389/fmolb.2026.1764620

**Published:** 2026-05-21

**Authors:** Mei Zhao, Lingxing Ouyang, Junjie Ma, Huimeng Zhu, Shengfang Zhu, Xuan Zhou, Bingyao Huang, Jun Yan

**Affiliations:** 1 Rehabilitation Medicine Department, Shenzhen Hospital of Integrated Traditional Chinese and Western Medicine, Shenzhen, China; 2 Acupuncture and Moxibustion Rehabilitation Department, Heyuan Traditional Chinese Medicine Hospital, Heyuan, China

**Keywords:** electroacupuncture, ferroptosis, HO-1, Nrf2, osteoarthritis

## Abstract

**Background:**

Electroacupuncture (EA) has shown beneficial effects in osteoarthritis (OA), but the underlying mechanism remains unclear. This study examined whether the effects of EA on OA are associated with changes in ferroptosis-related markers and Nrf2/HO-1 signaling.

**Methods:**

Rats were randomly assigned to the Control, OA, OA + ferrostatin-1, OA + EA, and OA + ferrostatin-1+EA groups. Gross cartilage appearance, paw withdrawal threshold, body weight, and knee joint width were evaluated. Cartilage injury was assessed by HE staining, Safranin O-fast green staining, and OARSI scoring. Inflammatory cytokines in serum and synovial fluid were measured by ELISA. TUNEL staining, immunofluorescence, biochemical assays, and western blot were used to assess apoptosis, ferroptosis-related markers, and Nrf2/HO-1 signaling in cartilage tissue.

**Results:**

Compared with the Control group, OA rats showed obvious cartilage damage, lower paw withdrawal threshold, reduced body weight gain, and increased knee joint width. Histological injury and OARSI scores were markedly increased in the OA group. Serum and synovial fluid levels of IL-6, IL-1β, and TNF-α were also elevated. In cartilage tissue, OA was associated with increased apoptosis, decreased SLC7A11, GPX4, SOX9, and GSH levels, and increased Fe^2+^ and ACSL4 levels. Nrf2 and HO-1 expression was increased in OA cartilage. Treatment with ferrostatin-1, EA, or the combined treatment alleviated these changes to different degrees.

**Conclusion:**

EA alleviated OA-related structural and inflammatory changes and was associated with reduced ferroptosis-related alterations and enhanced Nrf2/HO-1 signaling.

## Introduction

Osteoarthritis (OA) is the prevalent type of arthritis characterized by the degeneration of joint cartilage (Emami et al., 2023). It initiates with biochemical and cellular changes in the synovial joint tissues, leading to histological and structural alterations in the joints and eventually causing functional impairment (Yao et al., 2023). Recent perspectives on the pathogenesis of OA suggest that vascular dilation, bone remodeling, and disrupted bone turnover are influenced by various risk factors such as aging, obesity, and mechanical overload (Zhao et al., 2023). The goals of OA treatment include relieving joint pain and stiffness, preserving or increasing joint flexibility and stability, improving mobility and participation, and enhancing quality of life (Küçükdeveci, 2023). However, the current therapeutic prospects are insufficient in providing reliable pharmacological interventions and effective treatment approaches to address this increasingly prevalent issue (Sheng et al., 2023). Given the significant and expanding global burden, there is an urgent need for the development of novel treatment modalities.

In recent decades, acupuncture has gained increasing international attention (Ma et al., 2023). As a non-pharmacological treatment method, acupuncture has become increasingly important in pain management (Brinkhaus et al., 2020). By inserting fine metal needles into specific acupoints on the body and manipulating them, acupuncture has been proven to be an effective method for pain relief (Lin et al., 2022). Currently, electroacupuncture is extensively utilized in the clinical management of OA affecting the knee (Chen et al., 2021). In clinical settings, various electroacupuncture techniques have been extensively applied for the treatment of OA, not only alleviating pain and disability caused by OA but also reducing inflammation and the progression of OA pathogenesis, with equivalent or superior efficacy compared to conventional therapies. Furthermore, research on the mechanisms of electroacupuncture in relieving OA has mainly focused on improving pathological changes such as joint tissue histology, cartilage morphology, synovial inflammation, infiltration, and subchondral bone remodeling (Li et al., 2022). However, the exact mechanisms by which electroacupuncture improves cartilage damage remain unclear.

New research indicates that the onset of OA is controlled by various mechanisms of cell death and cytokine types (Yang et al., 2021). Ferroptosis, a newly discovered regulated cell death process, is distinguished by the iron-dependent buildup of lipid hydroperoxides that can reach lethal levels. However, the specific reversal of ferroptosis can be achieved through the use of ferroptosis inhibitors (Zhang S. et al., 2022). Recent studies have shown that iron-induced death of chondrocytes contributes to the progression of OA (Zhang X. et al., 2023). Previous research has demonstrated that chondrocytes undergo ferroptosis in inflammatory conditions, and inhibiting ferroptosis could alleviate chondrocyte damage (Guo et al., 2022). Therefore, targeting ferroptosis may be a promising approach for future OA treatment. However, the mechanisms of how electroacupuncture interacts with ferroptosis in OA are not yet clear.

Nrf2 is a transcription factor that is sensitive to changes in cellular redox status and has a vital role in controlling the expression of genes. It plays a critical role in regulating both basal and inducible gene expression. Specifically, Nrf2 can regulate the expression of antioxidant enzymes, thereby offering protection against oxidative damage (He F. et al., 2020). Studies have demonstrated that Nrf2 is involved in the regulation of the heme oxygenase-1 (HO-1) axis, which serves as an effective target for anti-inflammatory interventions (Saha et al., 2020). Wu J et al. reported that Stevioside, a compound found in sweeteners, alleviates OA by modulating Nrf2/HO-1/NF-κB pathway (Wu J. et al., 2023). Tan Z et al. reported that Echinacoside alleviates OA in rats by activating Nrf2/HO-1 pathway (Tan and Zhang, 2022). However, the mechanisms of electroacupuncture and Nrf2/HO-1 signaling pathway in OA are still unclear.

Based on the above background, it is plausible to hypothesize that the Nrf2/HO-1 pathway and ferroptosis may play a role in the therapeutic effects of electroacupuncture on the reduction of OA. To this end, we conducted *in vivo* experiments, established a rat model of OA, and treated it with electroacupuncture therapy and ferroptosis inhibitor ferrostatin-1 to explore the potential mechanism of electroacupuncture in OA treatment. Our study may provide new perspectives for further research on OA treatment.

## Materials and methods

### Animals

Thirty male Sprague-Dawley rats (200–250 g) were provided by Guangzhou Ruige Biotechnology Co., Ltd. and were randomly divided into 5 groups with 6 rats in each group. They were Control, OA, OA + ferrostatin-1, OA + EA, and OA + ferrostatin-1+EA groups. The OA model was established by intra-articular injection of 50 μL (equivalent to 3 mg) of 60 mg/mL sodium iodoacetate (Philpott and McDougall, 2020; Izumi et al., 2012). In the OA + ferrostatin-1 group, rats received intra-articular injections of 1 mg/kg of ferrostatin-1 on the second day after modeling (Wan et al., 2023), twice a week for 3 weeks. In the OA + EA group, rats underwent EA treatment on the second day after modeling, for 20 min each session, 5 days a week for 3 weeks. In the OA + ferrostatin-1+EA group, rats received intra-articular injections of 1 mg/kg of ferrostatin-1, followed by modeling, and then received EA treatment. The Control group received intra-articular injections of an equal amount of saline. The acupuncture points were ST36 (Zusanli), EX-LE5 (Neixiyan), and Heding. The rats were placed in a restrainer, and acupuncture needles (0.25 mm × 13 mm) were inserted at the designated points. Needle insertion depth was adjusted according to the anatomical location of each acupoint, with reference to previous studies (Tan et al., 2022; Huang et al., 2025). The needles were connected to an SDZ-II electronic acupuncture instrument. Sparse wave stimulation was administered at a frequency of 2 Hz and an intensity of 1 mA for a duration of 20 min (Li et al., 2020a; Zhang et al., 2019; Zhang W. et al., 2023). Behavioral changes and pain responses of the rats were observed once a week, and their body weight and knee joint width were measured. Anesthetized rats had their knee joint width measured using a caliper (N et al., 2016). After the rats were sacrificed, the changes in the color and size of the cartilage surface, as well as any ulceration, were observed and photographed. Blood and synovial fluid samples were collected.

### Paw withdrawal threshold (PWT) detection

The pain behavior assessment was conducted using von Frey filaments. Rats were housed in modular cages equipped with a metal mesh floor that enabled direct contact with the plantar surface of each hind paw. Once the animals had sufficient time to acclimate to the environment and exhibited reduced levels of exploratory behavior (approximately 10 min), von Frey filaments were used for measurement. A single von Frey filament was applied perpendicularly to the plantar surface of the hind paw on the same side, taking care to avoid contact with the footpad. The force was applied gradually until the filament bent. After the filament was kept in place for 3 s, if a positive response such as withdrawal, shaking, or licking of the hind paw was observed, the next lower-intensity filament was applied for further testing. If no response was elicited, the next higher-intensity filament was applied until the filament with a bending force of 25 g was reached (Philpott and McDougall, 2020; Li et al., 2020a; Sun et al., 2022; He L. et al., 2020).

### Hematoxylin eosin (HE) staining

The knee joint cartilage tissue was collected and fixed in a tissue fixative solution (G1101, Servicebio). The samples were then subjected to heat treatment at 60 °C for 1–2 h and subsequently paraffin-embedded and sectioned. The sections were dewaxed and rehydrated with distilled water. They were stained with Hematoxylin (G1003, Servicebio) for 5–10 min, followed by counterstaining with Eosin for 3–5 min. Sections were dehydrated using a graded series of ethanol (95%–100%) for 5 min at each concentration. Afterward, the sections were treated with xylene (10023418, Sinopharm Chemical Reagent Co., Ltd.) for 10 min, repeated twice, and finally mounted with neutral mounting medium (10004160, Sinopharm Chemical Reagent Co., Ltd.) for observation under a fluorescence microscope (BA210T, Motic).

### Safranin O-fast green staining and OARSI scoring

For observation of cartilage degeneration, Safranin O-fast green staining was performed. Rat cartilage tissue was embedded in paraffin and cut into 2 μm sections. Sections were baked at 60 °C for 12 h. After dewaxing, the sections were stained with Safranin O and counterstained with Fast Green according to standard procedures. The sections were mounted with neutral mounting medium and observed and photographed under a microscope. Based on the Safranin O-fast green staining results, cartilage damage was further evaluated using the OARSI scoring system.

### Enzyme linked immunosorbent assay (ELISA)

ELISA was utilized to evaluate inflammatory factors IL-6, TNF-α, and IL-1β levels in serum and synovial fluid. IL-6 (RX302856R, RUIXIN BIOTECH), TNF-α (RX302058R, RUIXIN BIOTECH), and IL-1β (RX302869R, RUIXIN BIOTECH) ELISA assay kits were applied to measure IL-6, TNF-α, and IL-1β according to the manufacturers’ instructions.

### TdT-mediated dUTP nick-end labeling (TUNEL)

Knee cartilage tissue apoptosis was detected using a TUNEL kit (G1502, Servicebio). Cartilage tissues were sectioned and baked at 60 °C for 30–60 min. Sections were dewaxed to water. Subsequently, each sample was treated with 100 μL of Proteinase K (G1205, Servicebio), added dropwise, and incubated at 37 °C for 20 min. An appropriate amount of membrane-breaking solution (G1204, Servicebio) was added dropwise to the tissues, and the tissues were fully infiltrated and processed for 20 min, and samples were similarly washed with PBS after membrane-breaking treatment. The samples were washed with PBS. For each sample, dropwise addition of 100 μL of 1×Equilibration Buffer was performed to cover the region of the sample designated for examination, followed by incubation for 10–30 min. TdT enzyme incubation buffer was prepared. The excess 1×Equilibration Buffer was removed by gently blotting with absorbent paper around the equilibrated area, and then 50 μL of TdT enzyme incubation buffer was added to the targeted tissues for examination. The incubation process was carried out for 60 min at 37 °C, while being protected from light. Nuclei were subsequently stained with DAPI (G1012, Servicebio) for 10 min at 37 °C, followed by three rinses with PBS for 5 min each. Finally, sections were sealed with neutral gum and subjected to observation using a fluorescence microscope.

### Immunofluorescence (IF)

IF was employed to assess co-localized expression of Nrf2, HO-1, SLC7A11, GPX4 and SOX9 in knee cartilage tissues. Sections were baked at 60 °C for 12 h and subsequently deparaffinized to water. Sections were then sequentially placed in 100%, 100%, 95%, 85% and 75% ethanol for 5 min at each concentration. To inactivate endogenous enzymes, 3% H_2_O_2_ was added for 10 min at room temperature. The sections were blocked with 5% BSA (BS114-100 g, Biosharp) for 60 min, followed by addition of Nrf2 (16396-1-AP, Proteintech), HO-1 (10701-1-AP, Proteintech), SLC7A11 (A13685, ABCLONAL), GPX4 (67763-1-Ig, Proteintech), and SOX9 (82630T, CST) and incubation at 4 °C overnight. Subsequently, secondary antibody (RC0086-23, Rutron) was incubated at 37 °C for 90 min, and the nuclei were stained with DAPI. Antifade mounting medium (G1401, Servicebio) was applied to seal sections. Images were captured by fluorescence microscopy.

### Biochemical detection

According to the instructions, the GSH (BC1175, Solarbio) assay kit and the Fe2+ (BC5415, Solarbio) assay kit were employed to determine the levels of GSH and Fe2+ in rat cartilage tissue.

### Western blot

Western blot was performed to determine ACSL4, Nrf2, and HO-1 levels in cartilage tissue. Total protein was extracted from cartilage tissue using RIPA (P0013B, Beyotime). After quantification with a BCA kit (BL521A, Biosharp), total proteins were separated by SDS-PAGE and transferred to PVDF membranes. The membranes were blocked with 5% skimmed milk and incubated overnight at 4 °C with primary antibodies against ACSL4 (22401-1-AP, Proteintech), Nrf2 (16396-1-AP, Proteintech), HO-1 (10701-1-AP, Proteintech), and GAPDH (60004-1-Ig, Proteintech). The membranes were then incubated with Goat Anti-Rabbit IgG (111-035–003, JACKSON) or Goat Anti-Mouse IgG (115-035–003, JACKSON). Signals were visualized using an enhanced chemiluminescence substrate (K-12045-D50, Advansta) and analyzed with imaging software (Chemiscope 6100, CLINX). GAPDH was used as the internal control.

### Statistical analysis

Statistical analysis was performed using GraphPad Prism 8.0. Data were presented as mean ± SD. Differences among groups were analyzed by one-way ANOVA followed by multiple comparisons. A value of *P* < 0.05 was considered statistically significant.

## Results

### Behavioral detection and characterization of rats

**FIGURE 1:**
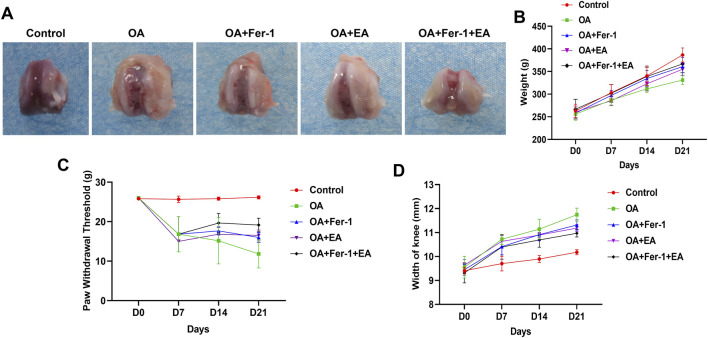
Behavioral detection and characterization of rats. **(A)** Observation of knee joint cartilage. **(B)** Changes in body weight of rats. **(C)** PWT detection. **(D)** Knee joint width changes of rats.

First, we established an OA model in rats and treated them with ferrostatin-1 and EA. Macroscopic examination of knee joint cartilage revealed cartilage damage and an irregular, rough surface in the OA group compared to the Control group. Treatment with ferrostatin-1 alone, EA alone, or the combination of ferrostatin-1 and EA improved the cartilage damage on the surface of the knee joint (Figure 1A). The PWT in the OA group significantly decreased compared to the Control group from day 7 to day 21. Treatment with ferrostatin-1 alone, EA alone, or the combination of ferrostatin-1 and EA increased the PWT in the rats (Figure 1C). Additionally, compared to the Control group, from day 7 to day 21, the body weight of the rats in the OA group gradually decreased, and the width of the knee joint increased. Treatment with ferrostatin-1 alone, EA alone, or the combination of ferrostatin-1 and EA partly restored body weight and decreased the width of the knee joint in rats (Figures 1B,D).

### Pathological analysis of cartilage tissue of knee joint in rats

**FIGURE 2:**
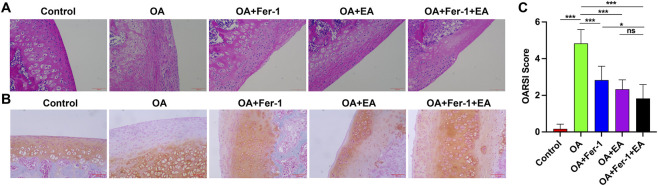
Pathological analysis of cartilage tissue of knee joint in rats. **(A)** HE staining of knee cartilage tissue. Magnification: ×100. **(B)** Safranin O-fast green staining of knee cartilage tissue. Magnification: ×100. **(C)** OARSI scores of cartilage lesions.

Next, we analyzed the pathological conditions of knee cartilage tissue in rats. HE staining showed that the cartilage surface of the OA, OA + ferrostatin-1, OA + EA, and OA + ferrostatin-1+EA groups was rough, fibrotic, and erosive, with reduced numbers of chondrocytes, blurred tidemarks, and a more incomplete structure than in the Control group. These changes were most obvious in the OA group (Figure 2A). In comparison to the Control group, the OA, OA + ferrostatin-1, OA + EA, and OA + ferrostatin-1+EA groups showed cartilage erosion. This change was most obvious in the OA group (Figure 2B). OARSI scores were also significantly increased in the OA group compared with the Control group, and were reduced after treatment with ferrostatin-1, EA, or the combination of ferrostatin-1 and EA (Figure 2C). Based on the current data, no clear additional benefit of the combined treatment over the single-treatment groups was observed.

### Levels of inflammatory cytokines IL-6, TNF-α, and IL-1β in serum and synovial fluid

**FIGURE 3:**
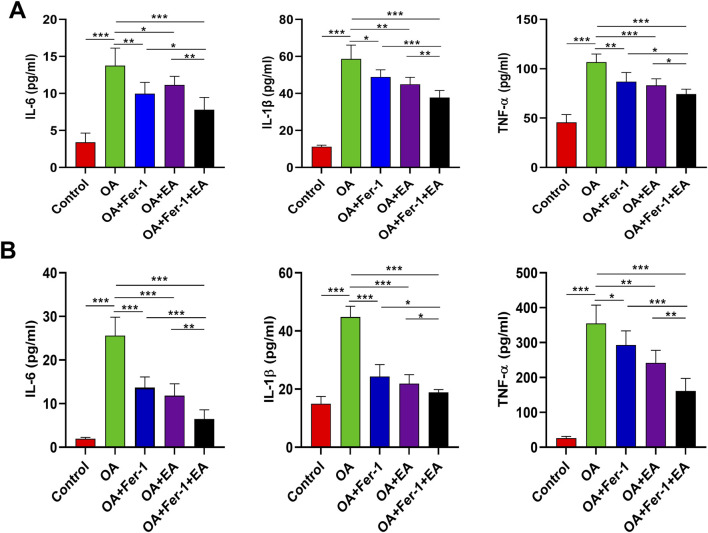
ELISA analysis of inflammatory cytokines in serum and synovial fluid. **(A)** IL-6, IL-1β, and TNF-α levels in serum. **(B)** IL-6, IL-1β, and TNF-α levels in synovial fluid. ns, no significance, *P < 0.05, **P < 0.01, ***P < 0.001.

In addition, inflammatory cytokine levels of IL-6, TNF-α, and IL-1β in the serum and synovial fluid of rats were significantly elevated in the OA group compared with the Control group. IL-6, TNF-α, and IL-1β levels were reduced with ferrostatin-1 and EA alone and in combination with ferrostatin-1 and EA (Figures 3A,B).

### Effects of acupuncture on apoptosis of knee cartilage and ferroptosis-related factors

**FIGURE 4:**
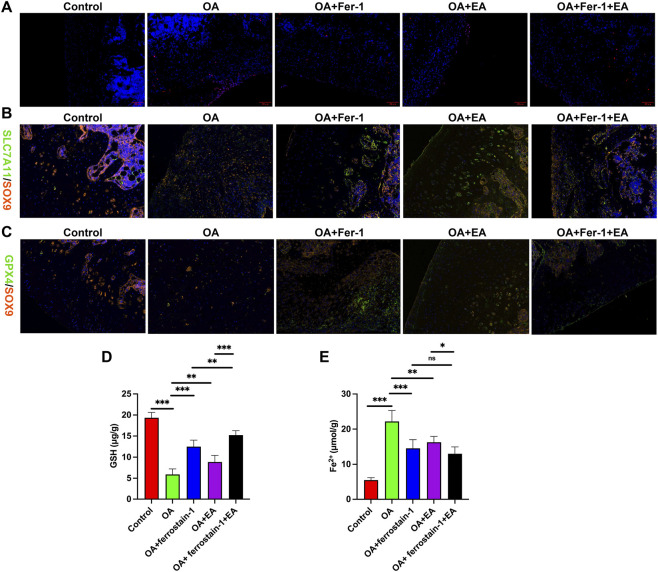
Effects of acupuncture on apoptosis of knee cartilage and ferroptosis-related factors. **(A)** TUNEL staining was utilized to determine apoptosis in knee joint cartilage tissue. Magnification: ×200. **(B)** IF staining of SLC7A11 and SOX9 in knee joint cartilage tissue. Magnification: ×200. **(C)** IF staining of GPX4 and SOX9 in knee joint cartilage tissue. Magnification: ×200. **(D)** GSH levels in cartilage tissue. **(E)** Fe^2+^ levels in cartilage tissue. ns, no significance, *P < 0.05, **P < 0.01, ***P < 0.001.

Furthermore, TUNEL staining revealed a significant increase in apoptosis in the cartilage tissue and surrounding areas of the knee joints in the OA, OA + ferrostatin-1, OA + EA, and OA + ferrostatin-1+EA groups compared with the Control group. Notably, the OA group exhibited particularly pronounced apoptosis, as shown in Figure 4A. We further detected the co-localized expression of SLC7A11, GPX4, and SOX9 in knee cartilage tissues using IF staining. In contrast to the Control group, the expression of SLC7A11, GPX4, and SOX9 was found to be downregulated in the knee cartilage tissue of the OA group. However, the expression of SLC7A11, GPX4, and SOX9 in knee cartilage tissues increased after treatment with ferrostatin-1 and EA alone and the combined use of ferrostatin-1 and EA (Figures 4B,C). Detection of GSH and Fe2+ levels in rat cartilage tissues showed decreased GSH levels and increased Fe2+ levels in the OA group compared with the Control group. After the administration of ferrostatin-1 and EA alone, as well as the combination of ferrostatin-1 and EA, there was an increase in GSH levels and a decrease in Fe2+ levels in rat cartilage tissues (Figures 4D,E).

### Effect of acupuncture on nrf2/HO-1 pathway-related factors

**FIGURE 5:**
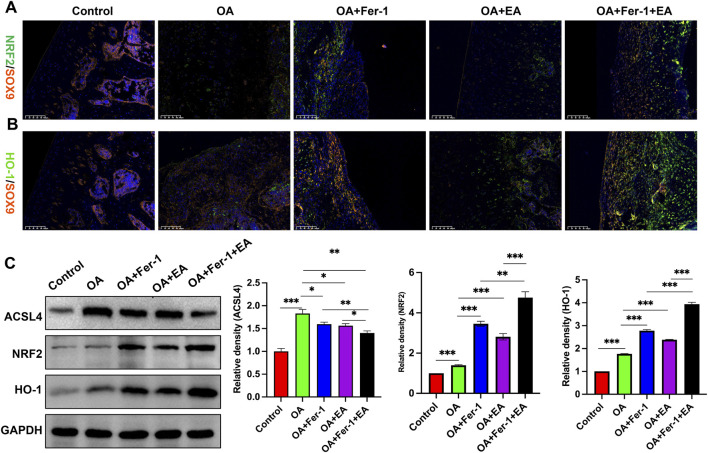
Effect of acupuncture on Nrf2/HO-1 pathway-related factors. **(A)** IF staining of Nrf2 and SOX9 in knee joint cartilage tissue. Magnification: ×200. **(B)** IF staining of HO-1 and SOX9 in knee joint cartilage tissue. Magnification: ×200. **(C)** Western blot analysis of ACSL4, Nrf2, and HO-1 in cartilage tissue. **P < 0.01, ***P < 0.001.

Finally, we further evaluated Nrf2, HO-1, and SOX9 levels in knee cartilage tissues using IF staining. As depicted in Figures 5A,B, in comparison to the Control group, the expression of Nrf2 and HO-1 in knee cartilage tissue was found to be upregulated in the OA group, while the expression of SOX9 was downregulated. However, the expression of Nrf2, HO-1, and SOX9 in knee cartilage tissues increased after treatment with ferrostatin-1 and EA alone and the combined use of ferrostatin-1 and EA. Western blot analysis showed that ACSL4, Nrf2, and HO-1 levels in the cartilage tissues of rats in the OA group were significantly higher than those in the Control group. After treatment with ferrostatin-1 alone, EA alone, or the combination of ferrostatin-1 and EA, ACSL4 expression was reduced, whereas Nrf2 and HO-1 levels were further increased (Figure 5C).

## Discussion

OA is a multifaceted process that involves various contributing factors such as mechanical load, inflammation, and metabolic factors (Yao et al., 2021). Current treatments, whether conservative or surgical, are designed to relieve symptoms and cannot delay or reverse the degenerative process of the joint (Wu C. et al., 2023). There is evidence that acupuncture could reduce pain, stiffness, and dysfunction in patients with OA, ultimately improving patient health (Luo et al., 2023). However, the mechanisms are unclear. In this study, we constructed a rat OA model and treated it with EA therapy and ferrostatin-1. We found that acupuncture was associated with activation of the Nrf2/HO-1 pathway, alleviation of OA, and reduced ferroptosis-related changes. To our knowledge, few studies have examined the relationship among acupuncture, ferroptosis, and the Nrf2/HO-1 pathway in OA, which highlights the novelty of the present study.

Acupuncture is a medical practice rooted in traditional Chinese medicine that involves the insertion of thin needles or the application of pressure to specific points on the body (Van Hal et al., 2023). There is evidence that acupuncture treatment combined with hyaluronic acid injection is more effective than hyaluronic acid injection alone in relieving pain and improving the function of the knee joint (Zheng et al., 2020). In addition, EA could significantly improve the clinical symptoms and quality of life of knee OA patients (Li et al., 2023). In this study, we first constructed a knee OA model by intra-articular injection of sodium iodoacetate, and then observed the effect of ferrostatin-1 and EA alone or combined with ferrostatin-1 and EA. We found that cartilage damage occurred on the knee surface of OA rats, the surface was rough and uneven, PWT decreased significantly, body weight decreased gradually, and knee width increased gradually. These conditions improved with ferrostatin-1 and EA alone and with ferrostatin-1 and EA together. In addition, our study showed that EA could reduce inflammatory factor levels in serum and synovial fluid of OA rats. Histological staining further showed that cartilage injury was accompanied by increased OARSI scores in the OA group, and these changes were attenuated after ferrostatin-1 or EA treatment. The combined treatment group also showed improvement, but no clear additional benefit over the single-treatment groups was observed in the present data. Taken together, our results suggest that EA exerted beneficial effects in OA, which may be related to the regulation of ferroptosis.

Iron is an essential element for many processes within cells. If cellular iron homeostasis is disrupted, iron will participate in the Fenton reaction (Fe2+ reduction of H2O2 to produce OH•), resulting in the formation of lipid peroxides and excessive accumulation of lethal ROS, thus leading to cell death (Yang and Stockwell, 2016; Dixon et al., 2012). Ferroptosis is a recently discovered form of cell death that is tightly regulated by iron metabolism, antioxidant processes, and lipid metabolism. It is known to play a crucial role in the development and progression of various diseases (Zhang Y. et al., 2022). Ferroptosis is manifested by the inactivation of GPX4, the regulatory core enzyme based on GSH depletion (Yang and Stockwell, 2016). GPX4 is responsible for transforming harmful lipid peroxides into harmless lipid alcohols (Ingold et al., 2018). Studies have shown that inhibiting ferroptosis could alleviate OA (Zhou et al., 2021). In this study, our findings suggested that EA may inhibit ferroptosis-related changes in OA. In addition to changes in GPX4, SLC7A11, GSH, and Fe2+, ACSL4 expression was increased in the OA group and decreased after ferrostatin-1 or EA treatment. This finding further supports the involvement of ferroptosis-related lipid metabolic changes in this process.

The Nrf2/HO-1 pathway serves as one of the vital endogenous defense systems in the body, and it represents the classical mechanism through which Nrf2 exerts its protective function (Li et al., 2020b). Nrf2 is a pivotal transcription factor that controls the expression of antioxidant enzymes. Its primary function is to safeguard against oxidative stress and protect tissues from damage (Chen et al., 2019). Indeed, the Nrf2/HO-1 signaling pathway is widely recognized as a protective molecular mechanism in situations of oxidative stress (Han et al., 2022). Guo Z et al. reported that deferoxamine alleviated OA by inhibiting ferroptosis and activating the Nrf2 pathway in chondrocytes (Guo et al., 2022). Wan Y et al. demonstrated that Baicalein inhibited chondrocyte ferroptosis by increasing the activity of AMPK/Nrf2/HO-1 signaling, thus alleviating the development of OA (Wan et al., 2023). In our study, Nrf2 and HO-1 expression was increased in the OA group and was further elevated after ferrostatin-1 or EA treatment. This pattern suggests that the upregulation of Nrf2/HO-1 in OA may reflect an endogenous compensatory response to oxidative stress, whereas its further enhancement after treatment may be associated with a protective effect. Taken together, these findings suggest that Nrf2/HO-1 activation in our model was mainly protective, while also showing compensatory features under OA conditions. This also suggests that the Nrf2/HO-1 pathway may be involved in the effects of EA. However, the present data do not establish a direct causal relationship between Nrf2/HO-1 activation and ferroptosis inhibition, which remains a limitation of this study.

## Conclusion

We preliminarily assessed the effect of acupuncture on OA and explored whether it was related to ferroptosis and the Nrf2/HO-1 pathway. Our findings suggest that acupuncture alleviated OA and was associated with activation of the Nrf2/HO-1 pathway and reduced ferroptosis-related changes. Our findings may help to improve the understanding of the molecular changes associated with OA. They may also provide a basis for further studies on EA in OA.

## Data Availability

The original contributions presented in the study are included in the article/supplementary material, further inquiries can be directed to the corresponding author.
